# XIAP inhibitors induce differentiation and impair clonogenic capacity of acute myeloid leukemia stem cells

**DOI:** 10.18632/oncotarget.2016

**Published:** 2014-05-26

**Authors:** Daniel Moreno-Martínez, Meritxell Nomdedeu, María Carmen Lara-Castillo, Amaia Etxabe, Marta Pratcorona, Niccolò Tesi, Marina Díaz-Beyá, María Rozman, Emili Montserrat, Álvaro Urbano-Ispizua, Jordi Esteve, Ruth M. Risueño

**Affiliations:** ^1^ Josep Carreras Leukaemia Research Institute. Barcelona, Spain; ^2^ Department of Hematology, Hospital Clínic de Barcelona. Barcelona, Spain; ^3^ Institut d'Investigacions Biomèdiques August Pi i Sunyer, Barcelona, Spain; ^4^ Department of Hematopathology, Hospital Clínic de Barcelona, Barcelona, Spain

**Keywords:** Acute myeloid leukemia, leukemic stem cell, new drugs, XIAP, Embelin

## Abstract

Acute myeloid leukemia (AML) is a neoplasia characterized by the rapid expansion of immature myeloid blasts in the bone marrow, and marked by poor prognosis and frequent relapse. As such, new therapeutic approaches are required for remission induction and prevention of relapse. Due to the higher chemotherapy sensitivity and limited life span of more differentiated AML blasts, differentiation-based therapies are a promising therapeutic approach. Based on public available gene expression profiles, a myeloid-specific differentiation-associated gene expression pattern was defined as the therapeutic target. A XIAP inhibitor (Dequalinium chloride, DQA) was identified in an *in silico* screening searching for small molecules that induce similar gene expression regulation. Treatment with DQA, similarly to Embelin (another XIAP inhibitor), induced cytotoxicity and differentiation in AML. XIAP inhibition differentially impaired cell viability of the most primitive AML blasts and reduced clonogenic capacity of AML cells, sparing healthy mature blood and hematopoietic stem cells. Taken together, these results suggest that XIAP constitutes a potential target for AML treatment and support the evaluation of XIAP inhibitors in clinical trials.

## INTRODUCTION

Acute myeloid leukemia (AML) is a hematological neoplasia characterized by an abnormal growth and differentiation of immature myeloid cells that accumulate in bone marrow and interfere with the production of normal blood cells [1]. In spite of some improvement in its therapy, a minority of adults with AML are eventually cured, a fact stressing the need for new therapeutic approaches.

AML is thought to be organized as a hierarchy of functionally heterogeneous cells that is ultimately sustained by a small fraction of leukemic stem cells (LSCs) with enhanced self-renewal capacity, impaired differentiation ability and increased drug resistance [2]. Among all AML blasts constituting the bulk tumor, only LSCs initiate and sustain the disease. As the balance between self-renewal and differentiation is tightly regulated and favours the former in LSCs, induction of terminal differentiation of LSCs would exhaust the LSC pool [3, 4]. Since differentiating therapy could induce higher chemotherapy sensitivity and limited life span of more differentiated AML blasts, combinatorial strategies of differentiating and cytotoxic agents with the capability to eliminate LSCs are a promising therapeutic approach, which could result in a significant improved outcome of AML patients [5].

Although global mechanisms that block differentiation and increase self-renewal of AML cells are poorly understood, biological changes induced by agents that differentiate AML cells to diverse lineages are already described. Bioinformatics studies provided us the gene expression profile associated to granulocyte differentiation of AML cells treated with all-trans-retinoic acid (ATRA). This drug-induced gene expression pattern constituted the therapeutic goal in the search of agents with AML differentiating capacity. Following this approach and using Connectivity Maps [6], we identified the XIAP inhibitor Dequalinium chloride (DQA). XIAP inhibition induced cytotoxicity and differentiation in AML cells. Moreover DQA treatment preferentially affected leukemic stem cells, suggesting that targeting XIAP may contribute to eradicate the LSC population thought to be responsible for refractoriness and relapse episodes in AML and supporting further validation in pre-clinical and clinical studies.

## RESULTS

In order to identify drugs that could induce myeloid differentiation in AML cells, a gene signature associated with ATRA-induced granulocytic differentiation of HL-60 AML cells was defined using public available gene expression profiles (GSE982). Differentially expressed probe sets were identified (Supplementary Table 1) and the given gene signature (Supplementary Table 2) was used to query and compare against the reference catalogue of gene expression profiles obtained after drug or other perturbagen treatment of cell lines using the Connectivity Map (http://www.broadinstitute.org/cmap/) [6]. The results were filtered at a P value <0.05; connectivity score > 0.75 in HL-60, and > 0.5 in PC3 and MCF7; and a concentration < 10 μM in all cell lines (Supplementary Table 3). Dequalinium chloride (DQA, CAS no. 522-51-0) was the only compound that met all the above-described criteria. DQA is an amphiphilic quinolinium derivative (Supplementary Figure 1).

**Figure 1:**
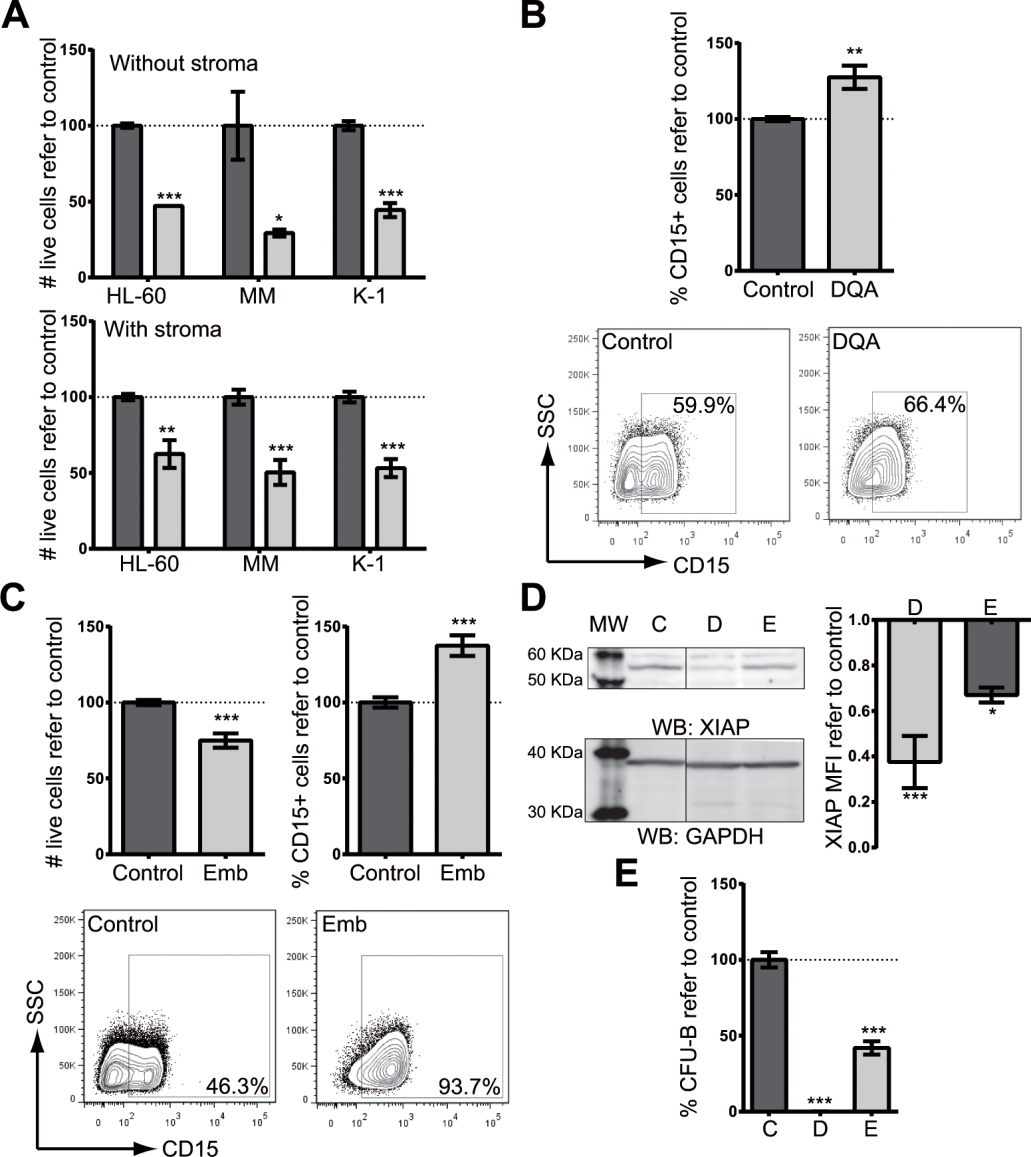
XIAP inhibitor treatment induces cytotoxicity and differentiation on AML cell lines A. Cytotoxicity in HL-60, MonoMac-1 (MM) and Kasumi-1 (K-1) AML cell lines resulting from treatment with 5 μM DQA for 48 h in the absence (upper panel) or presence of HS-5 stroma cells (lower panel). Y-axis: relative number of live cells as assessed by flow cytometry (7-AAD^−^). B. Up-regulation of CD15 surface expression, measured by flow cytometry in AML cell lines (HL-60, KG-1, MonoMac-1 and Kasumi-1) treated with 5 μM DQA. Data from all AML cell lines are presented combined. Frequency of CD15-positive population normalized against control-treated samples is represented. CD15 surface expression representative plot of HL-60 untreated (left) or treated with 5 μM DQA (right). C. HL-60, Kasumi-1, MonoMac-1 and KG-1 AML cells were treated with different concentrations of Embelin for 48 h. Cell viability (upper left panel) and CD15 surface expression (upper right panel) were measured by flow cytometry. Representative flow cytometry plot of HL-60 untreated (left) or treated with 10 μM Embelin (right). D. XIAP protein was detected by Western blot upon DQA (D) and Emb (E) treatment of HL-60 cells. GAPDH was used as loading control. MFI refer to GAPDH and vehicle-treated control is represented. E. HL-60 cells were treated for 18 h with 5 μM DQA (left) and 10 μM embelin (right). Colonies were counted at day 7. * p<0.05; ** p<0.005; *** p<0.0005. Error bars correspond to SEM.

We next studied the cytotoxic potential of DQA on well-characterized AML cell lines (HL-60, KG-1, MonoMac-1 and Kasumi-1). DQA induced cell death in all AML cell lines tested in a dose-response fashion after a 3-day treatment, achieving more than 50% of cell death at the highest concentration (5 μM) (Figure 1A). Since accumulating evidence shows that the bone marrow stromal environment might protect leukemia cells from chemotherapy-induced apoptosis, the cytotoxic potential of DQA was also tested on AML cell lines co-cultured with the HS-5 human bone marrow stroma cell line. Cellular viability of DQA-treated AML cells was impaired regardless the presence of HS-5 stroma cells (Figure 1A). Moreover, the proportion of cell death induced by DQA was similar regardless the presence of stroma cells, suggesting that the HS-5 stroma cells, that conferred chemoresistance to commonly used drugs such as ara-C and idarubicin [7], are unable to protect AML cell to the cytotoxic effect of DQA treatment. DQA was identified as a potential differentiation-inducer agent in our *in silico* screen. CD15 is up regulated in AML cells when differentiation is restored [8]. In all AML cell lines tested, DQA induced the upregulation of the CD15 surface marker (Figure 1B). These findings validated our *in silico* prediction of DQA as a differentiation-inducing drug of AML cells.

DQA has been identified as a XIAP inhibitor by its direct binding [9]. In order to confirm that XIAP inhibition was responsible for the cytotoxic and differentiation effects observed upon DQA treatment, a well-described XIAP inhibitor embelin was chosen[10]. As shown with DQA, embelin induced cytotoxicity and upregulation of CD15 surface expression (Figure 1C). In fact, both inhibitors reduced the amount of XIAP upon treatment (Figure 1D). Moreover, DQA and embelin treatment decreased the clonogenic capacity of AML cells (Figure 1E). These results suggest that XIAP inhibition overcomes the block in differentiation displayed by AML cells and reduces cell viability.

**Figure 2:**
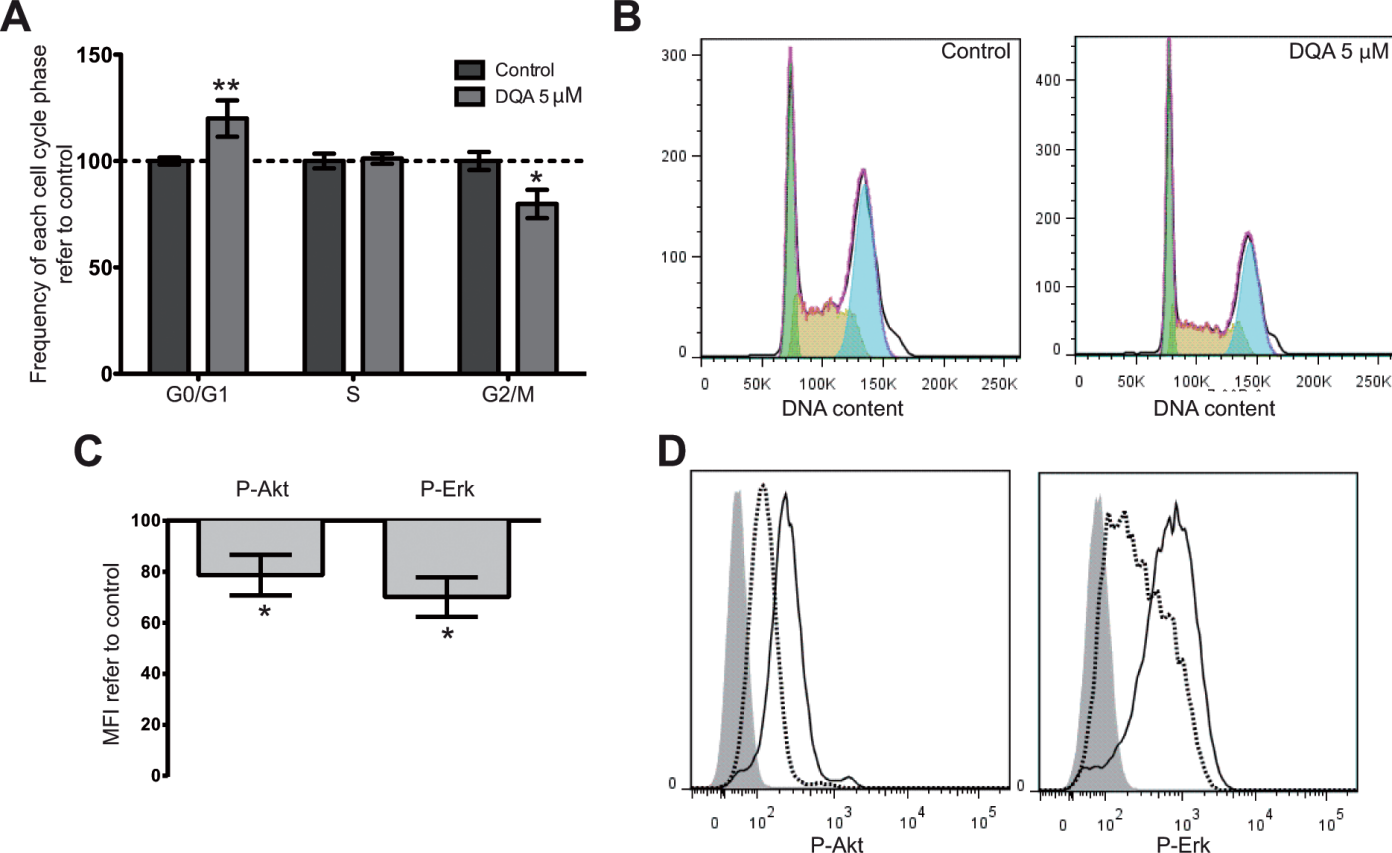
DQA treatment induces cell cycle arrest and downregulation of P-Akt, P-Erk and P-Stat3 HL-60, KG-1, MonoMac-1 and Kasumi-1 were treated with 5 μM DQA and cell cycle was analyzed by flow cytometry 48 h after treatment. A. Relative frequency of G0/G1, S and G2/M phases in control- vs. DQA-treated AML cells. Bars represent the mean value of all AML cell lines and error bars represent SEM. B. Representative DNA content flow profile of control- (left) and DQA-treated (right) HL-60 (green represents G0/G1 phase; yellow, S phase; blue, G2/M phase). P-Akt and P-Erk expression levels by flow cytometry in AML cell lines after treatment with 5 μM DQA for 24 h. C. Mean fluorescence intensity of each staining was normalized against vehicle-control treated sample and data from all AML cell lines tested is represented. D. Representative flow histograms of P-Akt (left), P-Erk (centre) and P-Stat3 (right) intracellular staining of DQA-treated HL-60 AML cells. Shadow, negative control; solid line, control treated sample; dashed line, DQA-treated sample. * p<0.05; ** p<0.005.

A way to promote differentiation is achieved through prevention of S-phase entry. This mechanism of action has been described for ATRA [11]. Similarly to ATRA, DQA treatment induced cell-cycle arrest in the G0/G1 phase whereas a reduction in G2/M phase was detected upon treatment of AML cell lines (Figure 2A and 2B).

**Table:** Characteristics of patient samples M, male; F, female. # × 10^9^/L

AML sample	Gender	Age (yr)	WHO subtype	WBC count#	% Blasts in PB	% Blasts in BM	Karyotype	Additional molecular features
#1	M	28	AML without maturation	143	98	90	46, XY	FLT3-ITD
#2	M	40	AML without maturation	52	66	80	46,XY	Biallelic CEBPA mutation
#3	F	34	AML with myelodysplasia-related changes	32	16	44	45,XX, -7	FLT3^wt^ NPM^wt^
#4	M	45	AML with t(6,9)	40	58	43	46,XY,t(6;9)(p23;q34)	FLT3-ITD
#5	M	48	AML with t(8;21)(q22;q22); RUNX1-RUNX1T1	54	5	24	46,XY,t(8;21)(q22;q22)	NPM^wt^ / FLT3 N/A
#6	M	61	AML with t(8;21)(q22;q22); RUNX1-RUNX1T1	21.4	51	89	45,X-Y,t(8;21)(q22;q22)[19]/46,XY[1]	FLT3^wt^ NPM^wt^
#7	F	58	AML with myelodysplasia-related changes	100.7	45	80	46,XX,del(5)(q23q33), t(8;9)(p11;q34)[20]	FLT3^wt^ NPM^wt^
#8	M	24	AML with myelodysplasia-related changes	7.1	83	30	46,XY[20]	FLT3^wt^ NPM^wt^
#9	M	49	AML with myelodysplasia-related changes	76.4	42	26	46-47,XY,del(5)(q22q34),del(6)(q22q25),del(7)(q22q23),-8,-9,add(11)(q23),+i(11)(q11),-16,+mar1,+mar2,+mar3[cp^8^].	FLT3^wt^ NPM^wt^
#10	M	22	AML with t(8;21)(q22;q22); RUNX1-RUNX1T1	20.4	83	69	45,X,-Y,t(8;21)(q22;q22)[17]/46,XY[3]	FLT3 ITD
#11	F	60	AML with myelodysplasia-related changes	218.1	68	36	48,XX,+8,+21[13]	FLT3^wt^ NPM^wt^

Several signaling pathways are misregulated in AML. Activation of Erk and Akt pathways [12, 13] have been considered as critical for the survival and/or proliferation of AML cells. In this context, DQA treatment was observed to reduce the amount of activated signaling molecules in all AML cell lines tested after intracellular staining of P-Akt and P-Erk (Figure 2C and 2D). These results correlate with the observed cytotoxic effect of DQA, which might at least in part be due to Akt and Erk downregulation.

**Figure 3:**
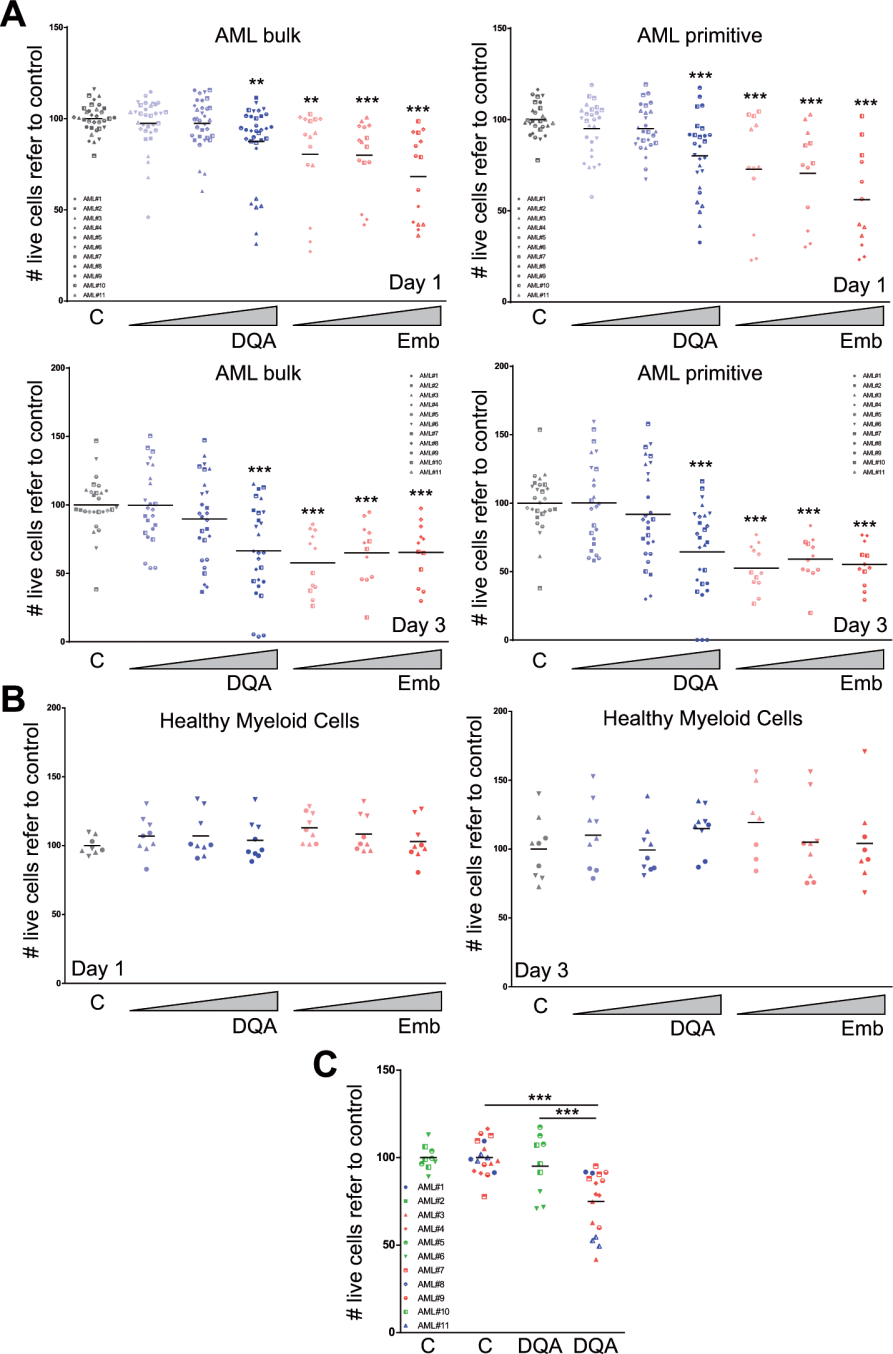
DQA and embelin treatment induces cell death in AML primary blasts by preferentially affecting LSC population and reduces clonogenic capacity AML primary blasts A. or healthy myeloid blood cells B. were treated with different concentration of DQA (0.05, 0.5, 5 μM) and embelin (0.1, 1, 10 μM). Cell viability was analysed at day 1 (upper panels) and 3 (lower panels) after treatment. Each symbol corresponds to a single AML patient sample, specified in the graph legend. Bulk population corresponds to AML blast cells and the primitive fraction corresponds to a CD34+CD38- blast population. C. Primary AML patient samples were treated for 24 h with 5 mM DQA. Cell viability was measured by flow cytometry (volumetric counts on live 7-AAD^−^ cells). Each symbol corresponds to an AML patient sample. Green, favourable risk group; blue, intermediate risk group; red, unfavourable risk group. * p<0.05; ** p<0.005; *** p<0.0005.

Next, the cytotoxicity of DQA and embelin treatment was evaluated in samples of patients with AML. The presence of these inhibitors reduced cell viability 24 and 72 h after treatment in the bulk AML population in a dose-dependent fashion (Figure 3A). Since the majority of the LSC population expresses the immature surface marker CD34 in the absence of CD38 [14], this marker combination (CD34^+^CD38^−^) was used to analyse the preferential effect of the drug on the LSC-enriched primitive population. Within the CD34^+^CD38^−^ population, the reduction in cell viability was higher in the presence of DQA or embelin compared to the remaining leukemic blast population (Figure 3A), suggesting that XIAP inhibitor cytotoxic effect is preferentially displayed on the stem-cell like or primitive population. Interestingly, no effect was detected when healthy myeloid blood cells were incubated with DQA or embelin (Figure 3B). Taking into account that XIAP expression has been described as a prognostic marker [15, 16], we analysed the cytotoxic effect of DQA treatment on the most primitive AML blast cell fraction within each prognostic group [17]. In concordance with protein expression data [15], intermediate and unfavourable risk groups were more sensitive to DQA treatment (Figure 3C).

**Figure 4:**
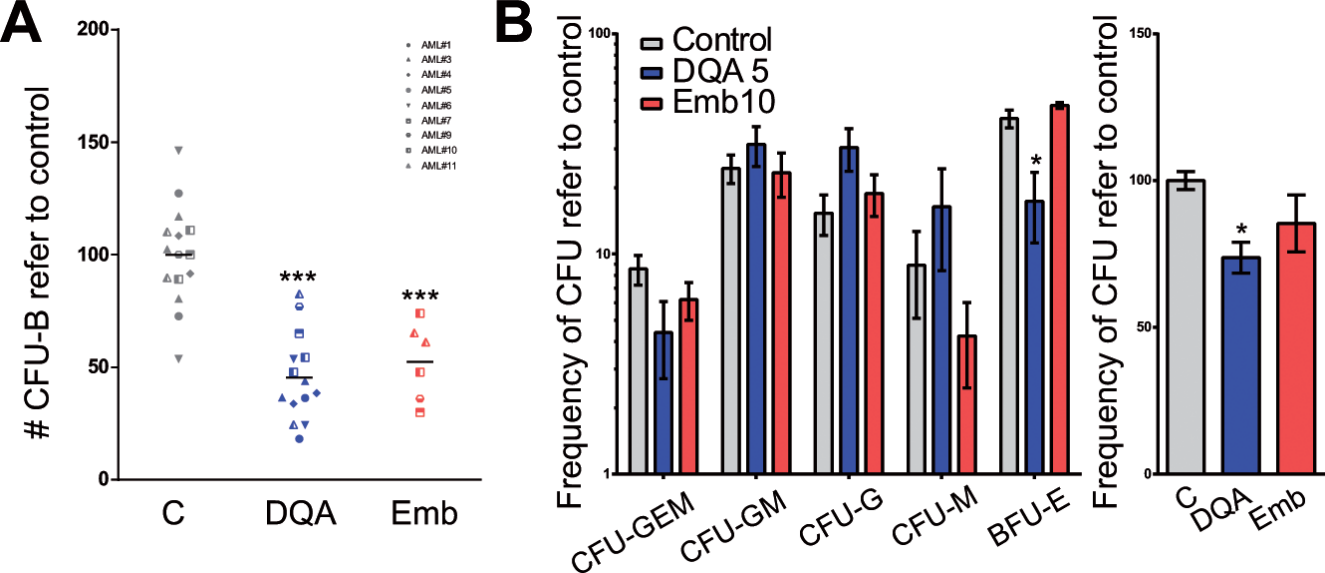
XIAP inhibitor treatment reduced the clonogenic capacity of AML cells with little effect on primitive healthy blood cells D. AML primary cells or E. lineage-depleted umbilical cord blood cells were treated with 5 μM DQA or 10 μM embelin for 18 h. Colonies were screened at day 14 based on morphological criteria. Each symbol represents primary sample. * p<0.05; ** p<0.005; *** p<0.0005.

Moreover, clonogenic capacity, which constitutes a direct measure of stem cell function [18], was reduced after DQA and embelin treatment of AML primary cells (Figure 4A). Treatment of lineage-depleted umbilical cord blood cells with DQA or embelin had little effect on the clonogenic capacity, as measured by the total number of colonies or the frequency of each subtype (Figure 4B). Taken together, these results suggest that XIAP inhibition preferentially impairs LSC functionality in AML.

## DISCUSSION

Although the notion that induction of differentiation by chemical agents in leukemic cells could reprogram the cells toward proliferation arrest and/or programmed cell death was established four decades ago [19, 20], only retinoic acid for the treatment of acute promyelocytic leukemia has revealed as a successful clinical application of this hypothesis. We performed an *in silico* screening seeking for FDA-approved drugs that produced a gene expression regulation similar to ATRA in HL-60 AML cells. A XIAP inhibitor, DQA, was identified and validated both in different AML cells lines and primary patient samples. XIAP inhibition also induced myeloid differentiation and cell death in AML cells. Interestingly, the clonogenic capacity was severely reduced upon treatment. Additionally, the most primitive fraction of AML blasts appeared to be more cytotoxic-sensitive to XIAP inhibition than more mature blasts. Interestingly, little effect was detected in healthy blood cells and primitive lineage-negative cord blood cells.

DQA is a classic broad bacteriostatic with anti-fungicidal and anti-protozoal activity [21, 22]. At a molecular level, DQA interrupts the interaction XIAP/Caspase-3 [9]. XIAP protein and mRNA levels have been correlated with chemoresistance [23] and poor clinical outcome in AML patients [15, 16], suggesting that XIAP may constitute an interesting therapeutic target for AML treatment. In fact, only most aggressive AML patient samples seemed to respond to DQA treatment. Phase I/II clinical trials evaluating the effect of a XIAP antisense oligonucleotide showed that this strategy seems to be effective when combined with chemotherapy in patients with AML refractory to a single induction regimen[24]. Interestingly, DQA-mediated cytotoxic effect seems to be stroma-independent. Microenvironment has been linked with primary LSC resistance to therapy where the niche signaling could regulate cell cycle and chemoresistance [25]. Here, XIAP inhibitors are described as AML differentiation inducer and, consequently, a reduction in clonogenic capacity and cell cycle arrest is produced. Similarly, other cell cycle arrest-inducing and pro-apoptotic drugs are effective in pre-clinical studies [26]. XIAP inhibitors already approved for clinical use, such as DQA, may be of great interest to explore their anti-leukemia effect and their potential therapeutic use in combination with conventional chemotherapy.

AML is hierarchically organized, with quiescent “stem-cell”-like cells (LSCs) at the apex, that have the ability to perpetuate themselves through self-renewal and generate more mature progeny through differentiation [2]. According with this model, the inability to eradicate LSCs represents the cause of relapse and therapeutic failure. Thus, effective tumor therapy will require eradication of these cells. Designing agents that can differentiate leukemia cells into non-dividing/non-malignant growth-arrested cells constitutes a new experimental approach for non-APL AML treatment. Here, XIAP inhibitors are described as AML differentiation inducer and, consequently, a reduction in clonogenic capacity and cell cycle arrest is produced. Therefore, there is a cessation of self-renewal capacity and XIAP may constitute a therapeutic target. Similarly, Bcl2L10 (another anti-apoptotic molecule) has also been described as a therapeutic target for AML and MDS [27]. Interestingly, an inhibitor against both Bcl-2 and Bcl-X, ABT-737, displayed anti-leukemia effect on AML [28]. Moreover, Pten/mTOR and the PI3K/Akt signalling pathway have been shown to be implicated in AML development and leukemia stem cell population maintenance [13, 29]. Here, XIAP inhibition reduced P-Akt levels in concordance with previous work and may explain the differential effect on the LSC population. XIAP inhibitors already approved for clinical use, such as DQA, may be of great interest to explore their anti-leukemia effect and their potential therapeutic use in combination with conventional chemotherapy.

In summary, our *in silico* screen for differentiation-inducing agents lead to the identification of DQA, an agent with a known capacity of XIAP inhibition. Interestingly, XIAP inhibitors proved to result in a significant cytotoxic and differentiating activity both in AML cell lines and primary samples, with a preferential effect on the immature leukemic stem-cell fraction and sparing healthy blood cells. These findings warrant further investigation in pre-clinical and clinical setting, in combination with currently used chemotherapy.

## MATERIALS AND METHODS

### Identification of genes related to myeloid differentiation with Connectivity Maps

Gene signature associated with ATRA-induced differentiation in HL-60 cells was obtained from GSE982. Raw files (.cel) were normalized and probe sets with a differential expression of at least 2-fold of change and p value <0.005 were chosen (Supplementary Table 1) using GenePattern software (Broad Institute Cancer Program; http://www.broadinstitute.org/cancer/software/genepattern/). The 49 top-ranking downregulated and 269 top-ranking upregulated probes during ATRA treatment were selected for *in silico* signature-based screening (Supplementary Table 2) (Connectivity Maps; http://www.broadinstitute.org/cmap/).

### AML cell lines and cell cultures

AML cell lines HL-60 (ACC-3)[30], KG-1 (ACC-14)[31], MonoMac-1 (ACC-252)[32] and Kasumi-1 (ACC-220)[33] were obtained from DSMZ (Deutsche Sammlung von Mikroorganismen und Zellkulturen) and the human stroma cell line HS-5 was purchased from ATCC (American Type Culture Collection). Experiments were performed within 6 months after receipt or resuscitation. AML cell lines were cultured in complete RPMI medium (PAA laboratories) supplemented with fetal bovine serum (Lonza), sodium pyruvate (Lonza) and/or non-essential amino acids (Lonza) according to manufacturers' recommendations. HS-5 cell line was cultured in complete DMEM medium (PAA laboratories) supplemented with 10% fetal bovine serum (Lonza). Primary AML blasts were cultured in IMDM (PAA laboratories) supplemented with 3% heat-inactivated fetal bovine serum (Lonza), 1x BIT (StemCell Technologies), 5 ng/ml IL3 (Peprotech), sodium pyruvate (Lonza) and β-mercaptoethanol (Sigma).

### Primary samples

Primary AML samples were obtained from patients diagnosed with AML at Hospital Clínic of Barcelona (Spain). AML diagnosis and classification was based on accepted WHO criteria. Main AML patient's characteristics are summarized in Table 1. Samples were obtained from bone marrow and mononuclear cells (MNCs) were isolated by Ficoll density gradient centrifugation (GE). All patients provided written informed consent in accordance with the Declaration of Helsinki, and the study was approved by the Ethics Committee of Hospital Clínic of Barcelona. Blood mature MNCs were isolated from healthy-donor buffy coats provided by Banc de Sang i Teixits (Barcelona, Spain). Umbilical cord blood MNCs were obtained after Ficoll density gradient centrifugation and were depleted for lineage marker-positive cells (Milteny).

### Drugs

Dequalinium chloride hydrate was obtained from Sigma-Aldrich, resuspended in ultrapure H_2_O (Sigma) and stored at −20°C at 5mM. Embelin was purchased from Enzo Life Science, resuspended in DMSO and stored at −20°C at 10 mM.

### Citotoxicity assay

Five-hundred thousand cells per ml were cultured in 96-well plates in complete medium. DQA or embelin were added at different concentrations. Co-culture experiments were performed seeding 30 × 10^3^ HS-5 cells with 1 × 10^5^ AML cells per 200 μl of complete medium. Cell viability was measured by 7-AAD (eBioscience) exclusion by flow cytometry and cell count was obtained by volume in a FACSVerse cytometer (BD). In co-culture experiments, AML cells were discriminated based on CD45 expression. Statistical analysis and IC50 determination were calculated in GraphPad (Prism software). FlowJo software (TriStar) was used for flow cytometry analysis.

### Myeloid differentiation

Cells were treated as indicated for cytotoxicity assays. Forty-eight hours after treatment, cells were stained with anti-human CD15-APC (BD) and surface expression of the antigen was analyzed by flow cytometry (FACSVerse, BD).

### Western Blot

HL-60 cells (10 × 106 cells) were incubated with 5 μM DQA or 10 μM embelin for 48 h. Cells were harvested and lysed in RIPA buffer. The protein extract was subjected to SDS-polyacrylamide gel (BioRad) electrophoresis and transferred to PVDF membrane (BioRad). Mouse anti-XIAP and goat anti-GAPDH antibodies were purchased from BD and Abcam respectively.

### Cell cycle analysis

Cells were cultured and treated with DQA as described above for experiments of differentiation induction. Twenty-four hours after treatment, cells were harvested, washed with PBS (Sigma), and fixed and permeabilized in 70% ethanol (Sigma) at 4°C. DNA content was stained with 7-AAD (eBioscience) and measured by flow cytometry (FACSVerse, Becton Dickinson). Data were analyzed using Watson cell cycle model in FlowJo 7.6.5.

### Intracellular staining

Cells were fixed with 2% formaldehyde (Applichem Lifescience), permabilized with 100% methanol (VWR BDH Prolabo), and stained with anti-Phospho-Akt (Thr308) clone C31E5E and anti-Phospho-Erk1/2 (Thr202/Tyr204) clone E10 from Cell Signaling Technologies following manufacturers' recommendations. RPE-coupled goat anti-mouse and anti-rabbit secondary antibodies (Life Technologies) were used and samples were acquired in a FACSVerse cytometer (BD). FCS files were analyzed in FlowJo software (TriStar).

### Clonogenic assay

Primary AML (50 × 10^3^) or HL-60 (1 × 10^3^) cells were treated for 18 h with the compound indicated and mixed with 1 mL of MethoCult H4034 Optimum (StemCell Technologies). Colonies were screened based on morphology and cellularity at day 5 (AML cell lines) or day 14 (primary AML cells).

## SUPPLEMENTARY FIGURE AND TABLES




