# Supplementary material for: XIAP inhibitors induce differentiation and impair clonogenic capacity of acute myeloid leukemia stem cells
Source: Oncotarget. 2014 May 26;5(12):4337–46. doi: 10.18632/oncotarget.2016 (PMC4147327; doi:10.18632/oncotarget.2016)
Supplement: Supplementary file 1 [file oncotarget-05-4337-s001.pdf]

XIAP inhibitors induce differentiation and impair clonogenic capacity of acute myeloid leukemia stem cells - Moreno-Martínez et al

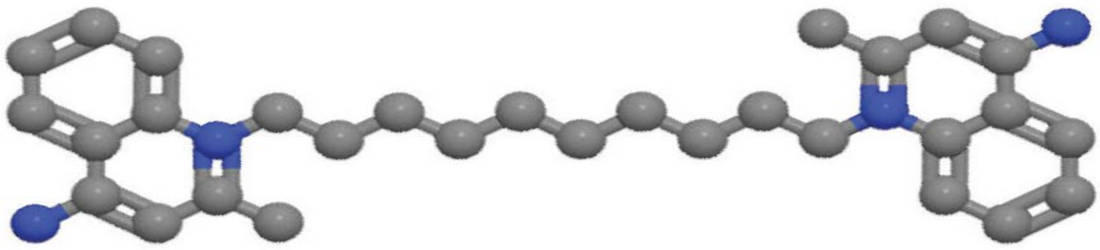

Supplementary Figure 1. Chemical structure of Dequalinium. Chemical structure generated in JMol software. Blue atoms: Nitrogen, Grey atoms: Carbon.
